# Effect of Metallic or Non-Metallic Element Addition on Surface Topography and Mechanical Properties of CrN Coatings

**DOI:** 10.3390/nano10122361

**Published:** 2020-11-27

**Authors:** Tatyana Kuznetsova, Vasilina Lapitskaya, Anastasiya Khabarava, Sergei Chizhik, Bogdan Warcholinski, Adam Gilewicz, Aleksander Kuprin, Sergei Aizikovich, Boris Mitrin

**Affiliations:** 1 Nanoprocesses and Technology Laboratory, A.V. Luikov Heat and Mass Transfer Institute of the National Academy of Science of Belarus, 15 P. Brovki str., 220072 Minsk, Belarus; kuzn06@mail.ru (T.K.); vasilinka.92@mail.ru (V.L.); av.khabarova@mail.ru (A.K.); chizhik_sa@tut.by (S.C.); 2 Faculty of Mechanical Engineering, Koszalin University of Technology, 2 Sniadeckich, 75-453 Koszalin, Poland; bogdan.warcholinski@tu.koszalin.pl (B.W.); adam.gilewicz@tu.koszalin.pl (A.G.); 3 National Science Center Kharkov Institute of Physics and Technology, 1 Academic str, 61108 Kharkiv, Ukraine; kuprin@kipt.kharkov.ua; 4 Research and Education Center “Materials”, Don State Technical University, 1 Gagarin sq., 344003 Rostov-on-Don, Russia; bmitrin@dstu.edu.ru

**Keywords:** AlCrN, CrON, topography, microparticles, roughness, AFM, elastic modulus, friction coefficient

## Abstract

Alteration of the phase composition of a coating and/or its surface topography can be achieved by changing the deposition technology and/or introducing additional elements into the coating. Investigation of the effect of the composition of CrN-based coatings (including AlCrN and CrON) on the microparticle height and volume, as well as the construction of correlations between the friction coefficient at the microscale and the geometry of microparticles, are the goals of this study. We use atomic force microscopy (AFM), which is the most effective method of investigation with nanometer resolution. By revealing the morphology, AFM allows one to determine the diameter of the particles, their heights and volumes and to identify different phases in the studied area by contrasted properties. The evaluation of the distribution of mechanical properties (modulus of elasticity *E* and microhardness *H*) on the surfaces of multiphase coatings with microparticles is carried out by using the nanoindentation method. It is found that the roughness decreases with an increase in the Al concentration in AlCrN. For the CrON coatings, the opposite effect is observed. Similar conclusions are valid for the size of the microparticles and their height for both types of coating.

## 1. Introduction

The application of multifunctional coatings is an effective way to control the surface properties of various products. A trend which has emerged in recent decades is the use of transition metal nitrides in the form of multicomponent systems, where each element of the additive enhances a specific function and simultaneously allows the achievement of excellent mechanical properties, corrosion and thermal resistance, wear and oxidation resistance [1,2,3,4]. The addition of components into the two-element transition metal–nitride system leads to the formation of various phases, grain refinement and various crystal lattices [5,6,7,8]. The addition of the third element to nitride systems expands their functional properties by changing the mechanical behavior of materials under the applied load depending on the element added [9,10,11,12,13]. This occurs due to the structural re-arrangement of multicomponent coatings that transform into nanocomposites, where both different crystal lattice polytypes and amorphous phases can be present [14,15,16,17]. Additionally, the surface microrelief of such coatings changes, which affects friction [18,19,20,21,22].

Chromium nitride is one of the most widely used coatings in industry. It provides high oxidation and corrosion resistance and good adhesion to steel substrates [1,23]. However, its hardness [24] and abrasion resistance are often insufficient. Alteration of the properties of CrN is achieved by alloying with metals and non-metals via the formation of ternary systems [25,26,27]. Al increases the thermal resistance, C and V reduce wear, the addition of B increases the hardness due to the grain refinement, and Si increases the oxidation resistance [1,5,28]. Due to the higher oxygen reactivity compared to nitrogen, even a small amount of oxygen in the presence of a growing metal nitride layer causes the formation of metal–oxygen ion bonds that appear in the matrix with a covalent metal–nitrogen bond and thus significantly changes the surface properties of CrN [29,30,31,32,33].

Thus, Al and O are the most effective and most known additives to CrN coatings [34,35]. The addition of Al to CrN increases its wear resistance at high temperatures due to formation of an oxide layer on worn surfaces [36]. The properties of AlCrN coating and the type of AlN phase lattice depend on the aluminum concentration in it. When Al content is below 75%, a cubic c-AlN phase is formed in AlCrN. An increase in the aluminum concentration to 80% promotes the formation of an h-AlN hexagonal phase [37]. Under mechanical loads and temperature, the transformation of the cubic c-AlN phase into the hexagonal h-AlN phase occurs spontaneously.

Technologists can effectively manipulate the surface properties of coatings by changing the phase composition of the coating by varying the technological parameters of the coating deposition (such as cathode current, bias, gas pressure) and introducing additional elements [4,38,39], which, to a great extent, depends on the microstructure of the surface—the size of microparticles (or micro-droplet phase), grains and the phase sizes on the surface [14,40,41].

One of the microstructural features of vacuum nitride coatings is that microparticles vary in size from 0.5 µm to several micrometers (according to other sources—a microdroplet phase) [42,43,44]. Particularly, microparticles are typical for the applied deposition method that is cathodic arc evaporation. Microparticles (or microdroplet phase) are important for tribological coatings, where they participate in the formation of “a modified tribolayer”. This layer is formed under the load from microparticles and from the upper plastically deformed surface layer of the coating. It is actively involved in the friction processes, especially under the conditions of so-called “green” processing without the use of cutting fluids [45,46].

The amount of microparticles is determined by the deposition mode and the cathode composition. In coatings manufactured by the cathodic arc evaporation method, the microparticles (microdroplets) can cover the surface with a continuous pattern [28]. Molten metal droplets ejected from cathode spots can move into the substrate and accumulate there as microparticles as well as agglomerates formed in plasma from atoms or ions. The microparticles are more plastic than the nitride phase that forms on the surface, due to the presence of the pure cathode metal in them [47]. The size and height of microparticles are always of interest in tribological coatings, since they allow the prediction of the characteristics of the contact between the coating and the counterbody and calculation of the real contact area.

For the effective implementation of new technological solutions within the framework of existing methods, it is necessary to rely on fundamental knowledge and the results of the microstructure and surface properties research with nanometer resolution [1,41,48]. Atomic force microscopy (AFM) allows for the versatile characterization of the coating surface geometry. AFM provides a multiscale (from scanning areas of 100 × 100 μm^2^ to 100 × 100 nm^2^ and less) surface visualization, allowing one to study both the microdroplet phase and submicron and nanometer grains of a smooth surface, by analyzing the contrast in properties to identify various phases among them [49,50]. By revealing the morphology, AFM makes it possible to determine not only the particles’ diameter but also their height and volume. More precisely, AFM allows one to measure all these characteristics of microparticles located “above the coating surface” but these microparticles are particularly important with respect to tribological contact. The height of the “above the coating surface” particles affects the value of the roughness, as well as the depth of the holes from chipping of microparticles. The effect of microparticle height on the roughness is higher because the microparticles are more common than the holes. Distribution of mechanical properties (elastic modulus *E* and microhardness *H*) on the surface of multiphase coatings with microparticles can be studied by nanoindentation (NI) [41,51].

The effect of the aluminum and oxygen concentration on the properties of AlCrN and CrON coatings was reported in [52,53,54,55] and [56,57], respectively. The structural, mechanical and tribological properties of the above coatings have been also studied by our group; for AlCrN, see [58,59,60], and for CrON, see [30,60,61,62]. It was determined that the microparticles affect the coatings’ properties [42,43,44]. The connection between the topography and mechanical properties of the surfaces of AlCrN and CrON coatings and their composition was essentially not studied. The effect of addition of the metallic or non-metallic element to the CrN coatings on the microparticle height and volume has not been studied previously; neither has the correlation between the friction coefficients and the geometry of the microparticles.

The aim of this research is to compare the effect of aluminum and oxygen concentration on the height, diameter and volume of the microparticles and to determine the correlation between the friction coefficient and the geometry of the microparticles in AlCrN and CrON coatings obtained by cathodic arc evaporation.

## 2. Materials and Methods

### 2.1. Coating Deposition

All coatings were synthesized by cathodic arc evaporation in systems summarized in Table 1. All cathodes were characterized by purity of 99.99%. The substrates, HS6-5-2 steel (ArcelorMittal, Katowice, Poland), with a diameter of 32 mm and a thickness of 3 mm, were finished to a roughness parameter *Ra* of around 0.02 µm. Ra was controlled by a contact profilometer, Hommel Werke T8000 (Hommelwerke GmbH, Villingen-Schwenningen, Germany). They were separately cleaned in an alkaline and ultrasonic bath, rinsed with deionized water and dried with warm air. The substrates were placed on a rotary holder parallel to the surface of the evaporated cathode, in the working chamber at a distance from the arc sources, depending on the deposition system used.

The vacuum chamber was evacuated to the base pressure and then heated to the required temperature. After reaching the desired temperature, the process of ionic etching of the substrate began, aimed at removing the oxygen adsorbed on the substrate surface, as well as the oxide layer. After the etching time had elapsed, a thin chromium layer was deposited on the substrate to improve the adhesion of the coating to the substrate. The deposition process was carried out in accordance with the parameters listed in Table 1.

The details of the three-step process of substrate preparation and coating formation in the working chamber, i.e., substrate ion cleaning, deposition the layer increasing adhesion to the substrate (adhesive layer) and the proper layer, are summarized in Table 1.

In the case of CrON coatings, a gas mixture (N_2_ + O_2_) with different relative oxygen concentrations, O_2(x)_ = O_2_/(N_2_ + O_2_)%, was used; see Table 1. Taking this into account, deposited coatings were labeled as follows: CrO(x)N. This means that, for example, CrO(20)N coating was obtained at a relative concentration of oxygen of 20%. In addition, the notation CrO(0)N coating has been simplified to CrN.

### 2.2. Coating Characterization

The structure of AlCrN and CrON coatings was analyzed by means of X-ray diffraction (XRD) in a conventional symmetrical Bragg–Brentano configuration (θ/2θ) with Co-Kα radiation and a DRON4 device (Burevestnik, Saint Petersburg, Russia).

The surface morphology and microstructure were evaluated by electron microscopy (JEOL JSM-5500LV, JEOL Ltd., Tokyo, Japan). The chemical composition of the microparticles in the coatings was analyzed by energy dispersive X-ray spectroscope (EDX) Link ISIS 300 (Link Analytical/Oxford Instruments, High Wycombe, UK). Detection accuracy of analyzed elements was within 3.0–5.0%.

The surface investigations of the coatings were carried out using Dimension FastScan atomic-force microscope (Bruker, Santa Barbara, CA, USA) in the PeakForce Tapping QNM (Quantitative Nanoscale Mechanical Mapping) mode. The standard silicon cantilevers of MPP-12120-10 (Bruker, Karlsruhe, Germany), NSC11 and CSG10 (Micromasch, Tallinn, Estonia) types and standard diamond probes of DRS_10 (TipsNano, Moscow, Russia) type were used. Particle Analysis was used to determine diameter, *d*, area, *S*, and height, *h*, of the microparticles and Bearing Analysis was used to determine the volume, *V,* of the microparticles in NanoScope Analysis software of Dimension FastScan AFM (Bruker, Santa Barbara, CA, USA). The Particle Analysis command defines some features of interest based on the height of pixel data. Particles may be analyzed alone or in quantities. Particles in this context are considered as conjoined pixels above or below a given threshold height. Bearing Analysis provides a method of plotting and analyzing the distribution of the surface height over a sample. The volume is defined above the bearing depth plane. To determine the particle content (%), the ratio of the total area of the particles in the field to the area of the field is calculated. AFM was used to analyze the surface topography with the determination of the roughness (*Ra* = arithmetic average of the absolute values of the surface height deviations measured from the mean plane; *Rq* = the standard deviation of the *Z* values within the investigated area). The forces between the AFM tip and the sample can be precisely controlled to prevent the movement of even loosely fixed microparticles [63,64]. The images of three areas were taken and analyzed on each sample. Every area contained fields of 60 × 60, 30 × 30 and 10 × 10 μm^2^. In the fields of 60 × 60 and 30 × 30 μm^2^, the influence of the microparticles on roughness was significant, and in the fields of 10 × 10 μm^2^, the roughness increased. Here, under the terms “height” and “diameter”, we refer to “the height or diameter of the particles above the surface of the coating”. The values *S* and *V* calculated in this work denote “the part of *S* and *V* of the microparticles above the coating surface”. Sometimes, the microparticles can be dispersed in the coatings but their presence on the surface significantly affects the friction properties and can be easily measured by AFM.

Microhardness (*H*) and elastic modulus (*E*) were measured using Hysitron 750 Ubi nanoindentation device (Bruker, Minneapolis, MN, USA) equipped with the Berkovich indenter with a curvature radius of 200 nm. The tip radius was calibrated by indentation into a fused quartz calibration sample. The values of *H* and *E* were calculated by the Oliver–Pharr formula from the experimental curves of continuous recording of the applied load–indentation depth. Two modes of nanoindentation (NI) curves were used. The first one involved load/unload curves; the second one involved progressive partial load/unload curves with partial unloading [65]. Entire load/unload curves were used for the calculation of the *E* and *H* mean values according to 9 curves at the load of 5 mN on a nominally flat surface. The indentation depth in this case did not exceed 110 nm, with a coating thickness of around 3 µm. Progressive partial load/unload curves with partial unloading were used to investigate the dependences of *E* and *H* on the penetration depth into coatings at the load of 0.1–10 mN. The maps of *E* and *H* for the upper coating layer with an average depth of indentation of around 20 nm were obtained by using the load/unload curves. The *E* and *H* maps with the size of 20 × 20 µm^2^ were obtained from 400 indentations at a load of 1 mN on the surface with the microparticles. 

The content of elements Al and O, peak intensity of c-CrN and h-AlN phases in the coatings, was a criterion for correlation analysis. The relationship between two parameters (geometrical characteristics of the microparticles or mechanical properties and the friction coefficient) with respect to the third constant feature—an increase in the content of non-metallic (oxygen) and metallic (aluminum) additives or a decrease in the content (peak intensity) of c-CrN and h-AlN phases—was determined. The correlation coefficient of the parameters *x* and *y* was determined by the following formula:

(1) $$r_{xy}=\frac{\sum^{\;}\left(x_{i}-\bar{x}\right)\cdot \left(y_{i}-\bar{y}\right)}{\sqrt{\sum^{\;}{\left(x_{i}-\bar{x}\right)}^{2}\cdot \sum^{\;}{\left(y_{i}-\bar{y}\right)}^{2}}}$$

where *y* corresponded to *Cfr* at a certain element content, and *x* alternately took the values of *E*, *H*, *d*, *h*, *S*, *V*, *Ra* and *Rq*.

## 3. Results and Discussion

**Figure 1:**
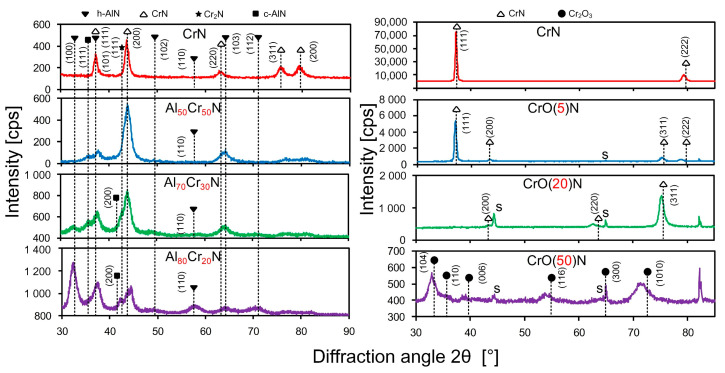
X-ray diffraction (XRD) diffraction patterns of AlCrN and CrON coatings.

Structural properties of the deposited CrN, AlCrN and CrON coatings measured by XRD are shown in Figure 1. The main phase in the CrN coatings obtained by two instruments is the CrN phase with a cubic crystal lattice (c-CrN). The main peaks in the CrN coating obtained by TINA are (200), (111), (220), (311). In the CrN coating obtained using the BULAT system, only one high-intensity diffraction line (111) is presented. It is more intense compared to line (222). It indicates a strong (111) texture (crystallography orientation) in this CrN coating. The main phase in the Al_50_Cr_50_N coating is c-CrN with (200) diffraction line. In the Al_50_Cr_50_N coating, the diffraction lines of the hexagonal AlN phase (h-AlN) appear from planes (101) and (103). In the Al_70_Cr_30_N coating, the intensity of the h-AlN phase lines increases. In the Al_80_Cr_20_N coating, the peak in the h-AlN phase becomes a preferred line. The intensity of the (111) c- CrN line in the coating CrO(5)N is the highest one but its intensity is lower than in the CrN coating obtained using the BULAT system. The diffraction line originating from planes (311) became the preferred line in the CrO(20)N coating. Only the Cr_2_O_5_ phase line is established in the CrO(50)N coating (Figure 1). The diffraction peaks from the Cr sublayer are absent.

The EDX analysis of the microparticles of coatings showed differences in the distribution of Al and Cr in AlCrN coatings and oxygen in CrON (Figure S1). This analysis is rather qualitative, aiming to show the differences in the element content between the microparticles and the surface. This difference in Al, Cr and oxygen content caused differences in the local distribution of the mechanical properties.

**Figure 2:**
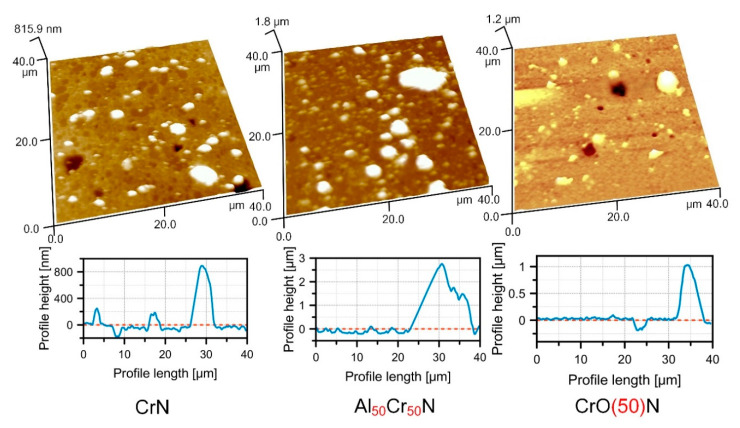
Three-dimensional atomic force microscopy (AFM) images and surface profiles of CrN, Al_50_Cr_50_N and CrO(50)N coatings with microparticles.

General assessment of the number of particles and their sizes on the CrN, AlCrN and CrON coating surfaces reveals the highest content and height for AlCrN, followed by CrN obtained at the TINA hardware, then CrON, and the smallest amount of microparticles with the lowest height for CrN was obtained using the BULAT unit (Figure 2). A significant difference was found in the amount (8.3% and 1.4%) and size (1.9 and 1.3 µm) of the microparticles in the CrN coatings deposited by either hardware. The XRD analysis demonstrated that the preferred orientation of the cubic CrN phase (c-CrN) in the coating obtained by TINA is (200). There are more other peaks of (111), (311), (220), whose intensities are significant in comparison with (200). The main peak of the c-CrN phase in the CrN coating obtained using BULAT is (111) and there is only one other peak of (222), which is insignificant in comparison with (111). In addition, the width of the peaks in the former coating relative to their width is significantly higher than in the latter. This qualitatively indicates a higher crystallinity degree in the second coating. The difference in the textures of the c-CrN phase can be explained by the different rates of atom and ion flow to the surfaces of the CrN coatings. For TINA, this flow was significantly higher due to the higher nitrogen pressure (more than two times) and shorter distance between the surface and the cathode. Etching was carried out in hardware in order to clean it. The time of cleaning for the first coating was almost three times longer, which could prepare a larger number of crystallization centers on the surface. Together, this resulted in more microparticles in the CrN coating produced by TINA. Based on the common statement about the effect of roughness on the friction coefficient (*Cfr*) and on the basis of this preliminary estimate, one could assume that the lowest values of *Cfr* should be on CrN obtained using the BULAT device, then slightly higher for CrON, succeeded by CrN obtained using the TINA unit, and finally, one should expect the highest *Cfr* on AlCrN.

**Figure 3:**
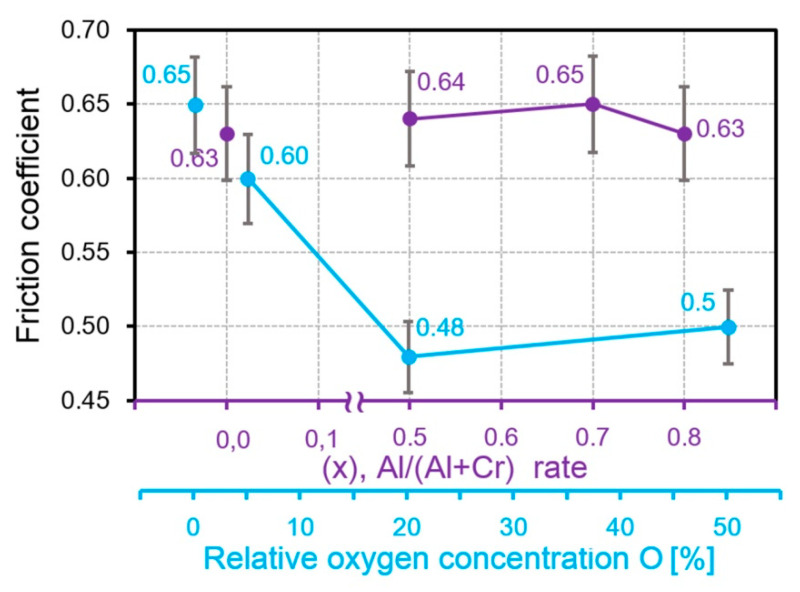
The dependences of the friction coefficient from the addition content in the CrN coatings.

The experimental results disagree with this assumption, since the phase composition, mechanical properties and surface microgeometry have to be taken into account simultaneously. Figure 3 shows the dependences of *Cfr* from the Al content in AlCrN and from the oxygen content in CrON coatings, constructed on the basis of data obtained in [28,51] under the same conditions of sliding friction without lubrication.

**Figure 4:**
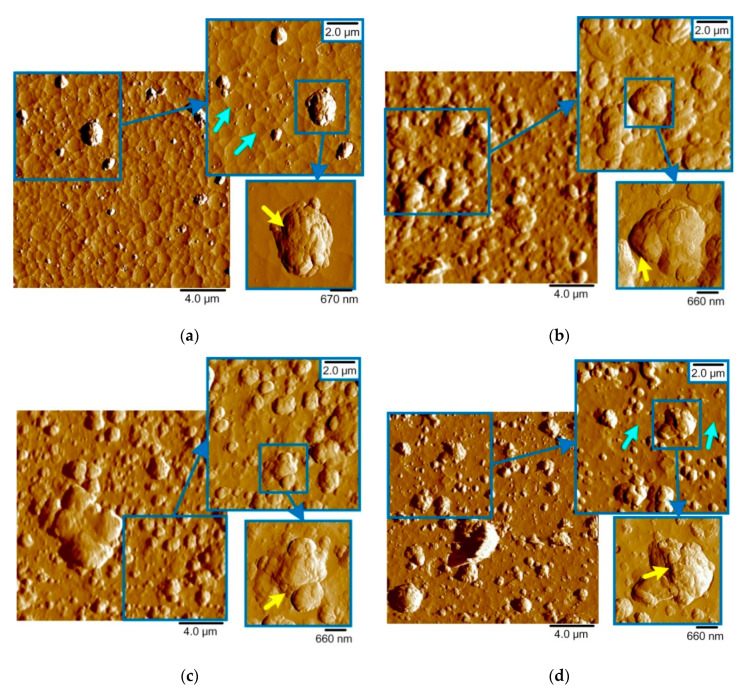
Two-dimensional AFM images of the AlCrN coatings, area 20 × 20 µm^2^: (**a**) CrN; (**b**) Al_50_Cr_50_N; (**c**) Al_70_Cr_30_N; (**d**) Al_80_Cr_20_N.

**Figure 5:**
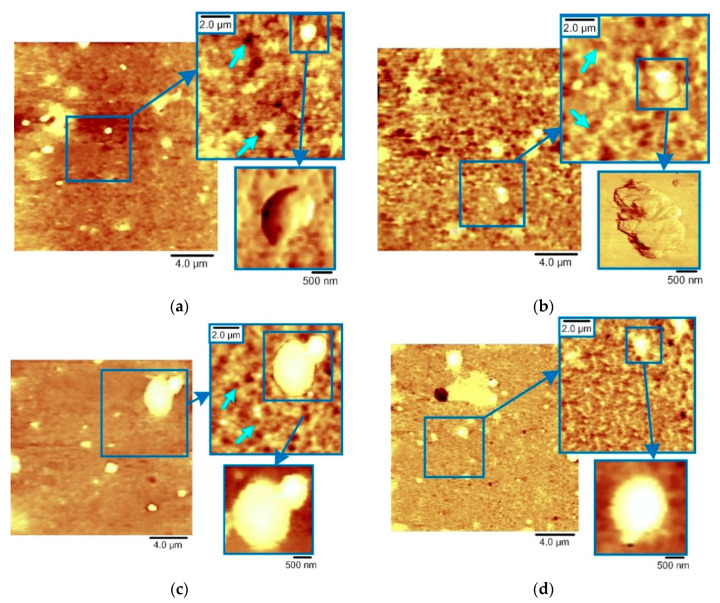
Two-dimensional AFM images of the CrON coatings, area 20 × 20 µm^2^: (**a**) CrN; (**b**) CrO(5)N; (**c**) CrO(20)N; (**d**) CrO(50)N.

The AFM capabilities make it possible to reveal the details of a significant difference in the morphology of the coating surfaces (Figure 4 and Figure 5). Cathodic arc evaporation forms a “smooth” coating surface consisting of connected cells (Figure 4a, blue arrows). The “ribs” of the cells are the protruding edges of the crystallites. The CrN coating obtained by the TINA unit demonstrates cell diameters of 0.5–2 µm. For the CrN coating obtained by the BULAT system, the cell diameter is approximately two times lower and is in the range of 0.2–1 µm (Figure 4a).

The surface images shown in Figure 4 are presented in the Error mode. This channel produces a map of the peak force measured during the scanning process. Because the PeakForce QNM mode uses the peak force as the feedback signal, this channel is essentially the Peak Force Setpoint with the error. It is recorded simultaneously with the topography. In this mode, one can better observe the boundaries between the layers in the microparticle images. The height scale corresponds to the real topography of the microparticles. The use of the Topography mode in the illustration of AlCrN microparticles, due to their height, gives the image “flare”, i.e., very tall particles look white and uniform and there is no way to show their internal microstructure.

The microparticle shapes in AlCrN and CrON coatings are different. In the AlCrN coatings, there are layered particles of irregular shape. The layers in the microparticles are marked in Figure 4 by yellow arrows and in Figure S2 by violet and yellow arrows. Their morphology shows that they were formed by the merging of gradually layering flat islets. The long axes of these flat islets are oriented differently with respect to the coating surface plane (Figure 4). With an increase in the Al content, the amount of microparticles increases. In CrON, both irregular, differently oriented and layered particles and regular spherical particles are observed. The correct spherical shape of such particles indicates that it is unlikely that they were formed by multiple hits of clusters of already grouped atoms outside the coating. It is possible that the regular spherical particles were formed as a result of the crystallization of single microdroplets in the working gas environment (Figure 5).

**Figure 6:**
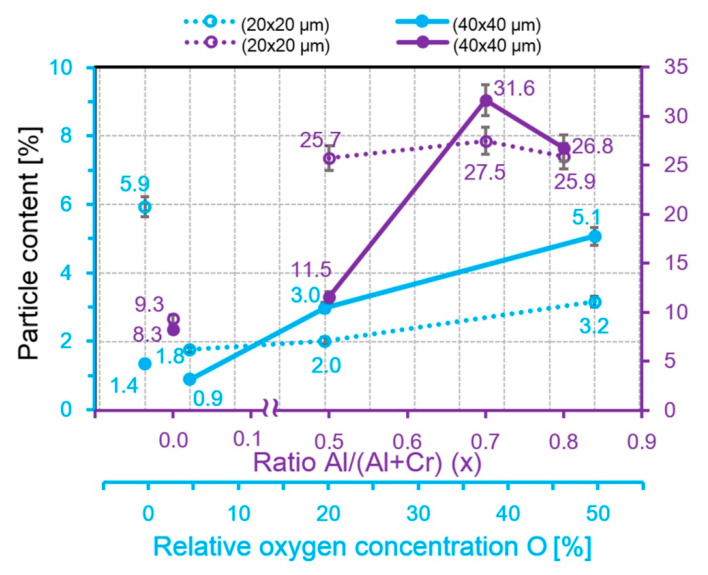
The dependences of the particle content from the addition content in the CrN coatings.

The percentage of particles in the AlCrN coating is ten times higher than that in the CrON coating (Figure 6). Assessing the geometric parameters of the microparticles, we used two sizes of AFM images, namely 40 × 40 µm^2^ and 20 × 20 µm^2^. The 40 × 40 µm^2^ field allows for a better assessment of the total amount of the microparticles, and better accuracy in determining the diameter and height of the microparticles is achieved using 20 × 20 µm^2^ fields. The dependences of the microparticle content on the oxygen amount in the CrON coating in the fields of 40 × 40 µm^2^ and 20 × 20 µm^2^ are almost linear (Figure 6). For the AlCrN coatings, the dependence of the content of the microparticles from the content of aluminum in the fields of 20 × 20 µm^2^ is close to linear, and in the fields of 40 × 40 µm^2^, it is non-monotonic, with a maximum at 70% Al. Such a difference in the form of the dependences of the particle content from the amount of Al in the AlCrN coating at different scales of fields shows the heterogeneity of the distribution of the microparticles and the importance of using two sizes of fields in their study.

**Figure 7:**
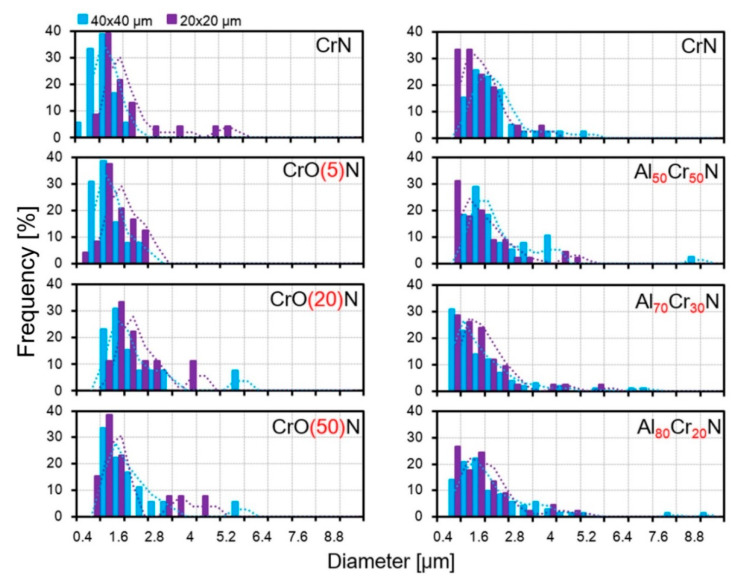
Histograms of the microparticle diameters of the AlCrN and CrON coatings in the fields of 40 × 40 μm^2^ and 20 × 20 µm^2^.

**Figure 8:**
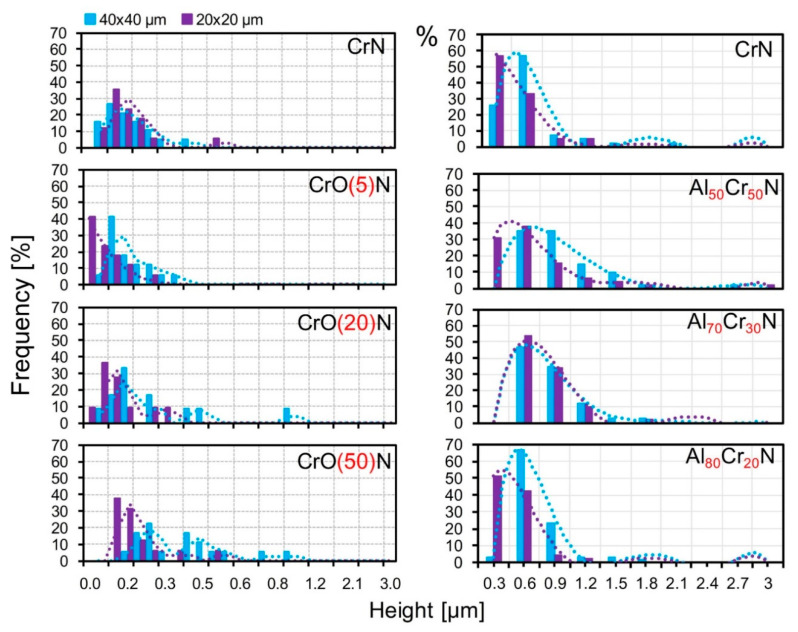
Histograms of the microparticle heights of the AlCrN and CrON coatings in the fields of 40 × 40 µm^2^ and 20 × 20 µm^2^.

It is difficult to perceive any difference for the CrO_20_N and CrO_50_N coatings by their distribution by diameter (Figure 7), but this can be identified by the average values. The microparticle distribution by height in the AlCrN and CrON coatings is shown in Figure 8. The higher sensitivity of the microparticle height to both the content of additives and the scale of the investigated microstructure is clearly manifested. The differences in the most frequently encountered intervals of the microparticle heights are much more significant than those in the diameters of microparticles (Figure 8). A difference in the particle heights in different fields of 40 × 40 μm^2^ and 20 × 20 μm^2^ in the distribution of heights is not detected for Al_70_Cr_30_N but is clearly seen from the average values.

**Figure 9:**
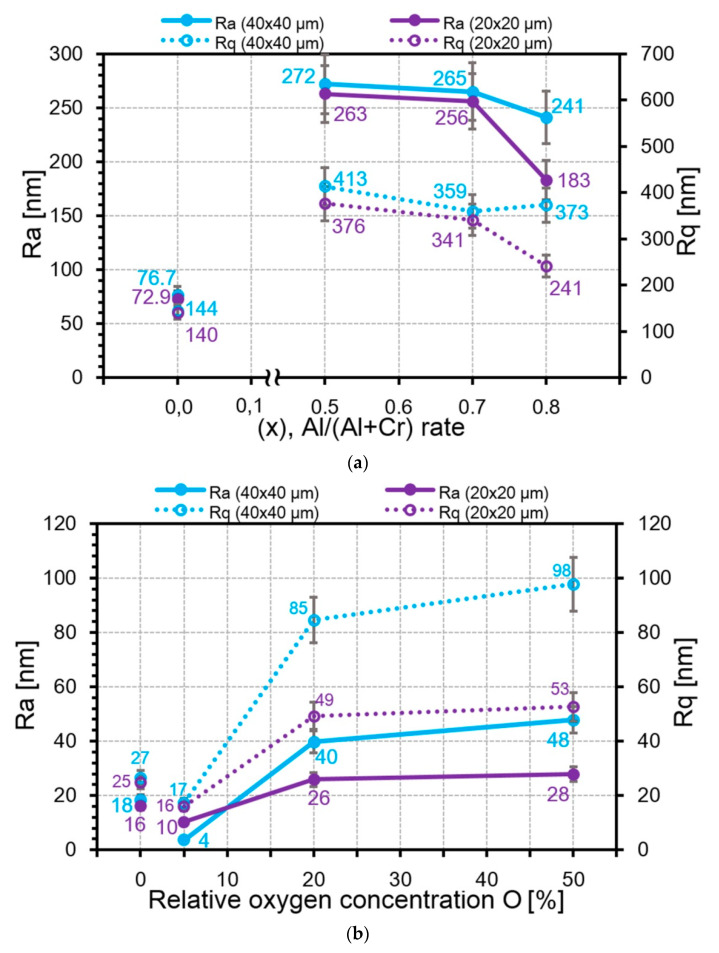
The dependences of *Ra* and *Rq* from the element concentration: (**a**) aluminum in the AlCrN coatings; (**b**) oxygen in the CrON coatings.

Roughness quite accurately reflects the different tendency in the microparticle height change of the AlCrN and CrON coatings (Figure 9), which is in good agreement with previous work [35]. For the AlCrN coatings, *Ra* decreases from 263 nm for Al_50_Cr_50_N to 183 nm for Al_80_Cr_20_N in the field of 40 × 40 μm^2^. In the field of 20 × 20 μm^2^, the height difference is smoother, from 272 nm for Al_50_Cr_50_N to 241 nm for Al_80_Cr_20_N. For the CrON coatings, the reverse process occurs: *Ra* increases when the oxygen content increases from 10 nm for CrO(5)N to 28 nm for CrO(50)N in the field of 40 × 40 μm^2^, and from 4 nm for CrO(5)N to 48 nm for CrO(50)N in the field of 20 × 20 μm^2^.

**Figure 10:**
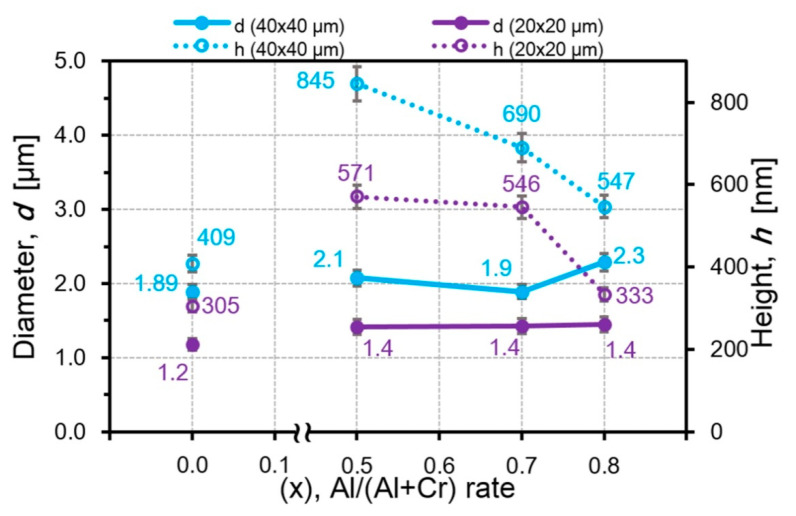
The dependences of the particle diameter and height from the aluminum content in the AlCrN coatings.

**Figure 11:**
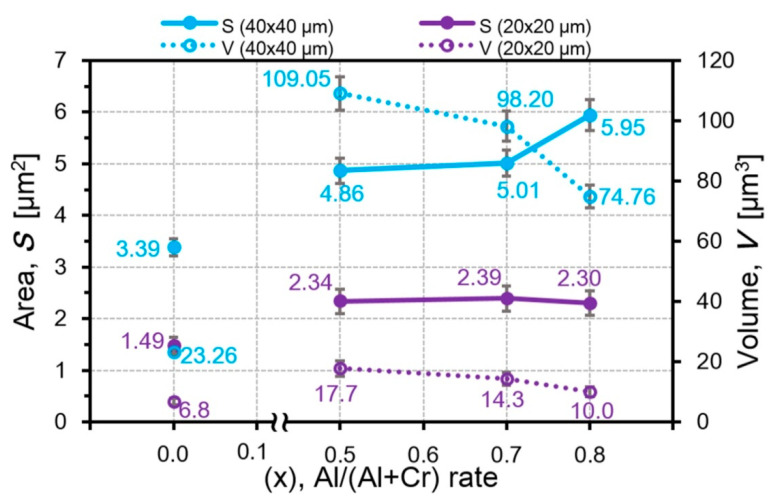
The dependences of the particle area and volume from the aluminum content in the AlCrN coatings.

Comparison of the height and diameter dependences from the Al amount in the AlCrN coatings clearly shows high sensitivity of the particle height to the Al content in contrast to the particle diameter. The particle diameter remains almost unchanged in the fields of 40 × 40 μm^2^ and does not change at all in the fields of 20 × 20 μm^2^ (Figure 10). In the AlCrN coatings, with an increase in the Al amount, the particle heights decrease from 845 nm for Al_50_Cr_50_N to 547 nm for Al_80_Cr_20_N in the fields of 40 × 40 μm^2^ and from 571 nm for Al_50_Cr_50_N to 333 nm for Al_80_Cr_20_N in the fields of 20 × 20 μm^2^ (Figure 10). Since the diameter of particles in the AlCrN coatings practically does not change with the increase in the Al amount, a decrease in the volume of the microparticles in the field of 20 × 20 μm^2^ from 17.7 μm^3^ for Al_50_Cr_50_N to 10.0 μm^3^ for Al_80_Cr_20_N is associated with a decrease in their heights (Figure 11).

**Figure 12:**
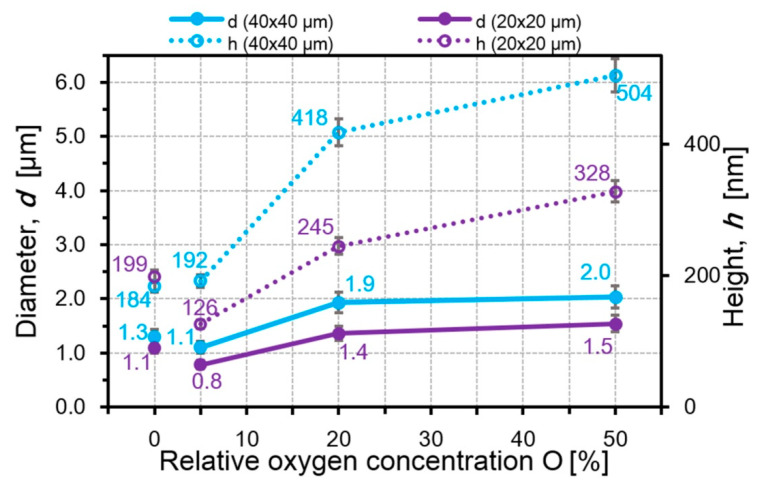
The dependences of the particle height and diameter from the oxygen content in the CrON coatings.

**Figure 13:**
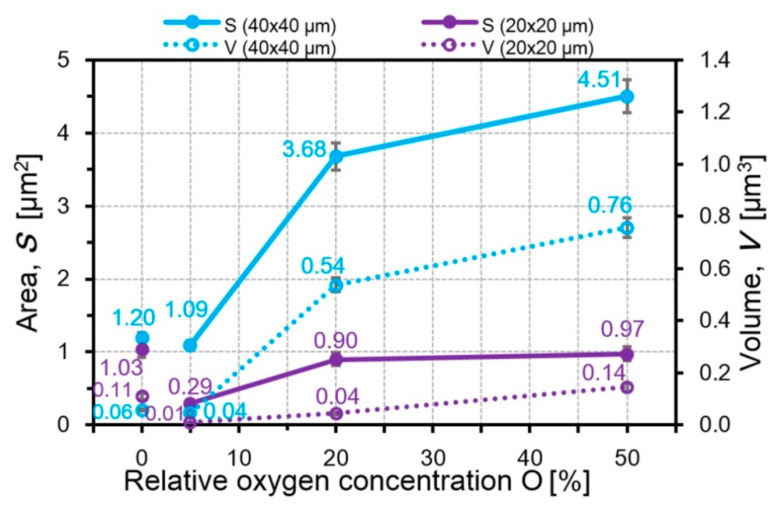
The dependences of the particle area and volume from the oxygen content in the CrON coatings.

However, the decrease in their total volume is possible not only with a constant diameter and decrease in the height of the particles. Even in the case when the particle diameter increases from 1.9 for Al_70_Cr_30_N to 2.3 µm for Al_80_Cr_20_N, the decrease in the particle height from 690 to 547 nm leads to the decrease in the particle volume in the field of 40 × 40 μm^2^ and the volume decreases from 98.2 μm^3^ for Al_70_Cr_30_N up to 74.7 μm^3^ for Al_80_Cr_20_N (Figure 11). In the CrON coatings, the particle diameter increases roughly twice (in CrO(20)N and CrO(50)N) with an increase in the oxygen amount but the particle height increases much more, around 2.5 times with respect to CrO(5)N (Figure 12). This leads to the growth of *S* and *V* of the microparticles (Figure 13). The largest increase in the volume of particles for the CrO(50)N coating was 0.75 μm^3^. 

When estimating the effect of additives on the amount of microparticles and the microparticle geometry, the AlCrN and CrON coatings should be compared within the groups, given that, due to the different deposition parameters (bias voltage, arc current, distance to the sample, pressure in the chamber) in the TINA and BULAT equipment, even the CrN coatings have significantly different amounts of microparticles.

Thus, the CrN coating obtained via TINA contains 9.3% of particles, *Ra* of 77 nm, *Rq* of 144 nm, average diameter of 1.9 µm, average height of 409 nm, average area of 3.39 μm^2^ and average volume of 1.49 μm^3^. The CrN coating obtained via BULAT contains 5.9% of particles, *Ra* of 18 nm, *Rq* of 18 nm, average diameter of 1.3 µm, average height of 184 nm, average area of 1.2 μm^2^ and volume of 0.1 μm^3^.

The microparticles on the surfaces of the cathodic arc deposited coatings significantly depend on the modes of the technological process [66]. Collisions between atoms or ions become more frequent under the influence of high pressure, and during the formation of the AlCrN coatings, it is almost twice as high as compared to CrN, which leads to the formation of agglomerates of a larger number of atoms before deposition on the substrate [28].

**Figure 14:**
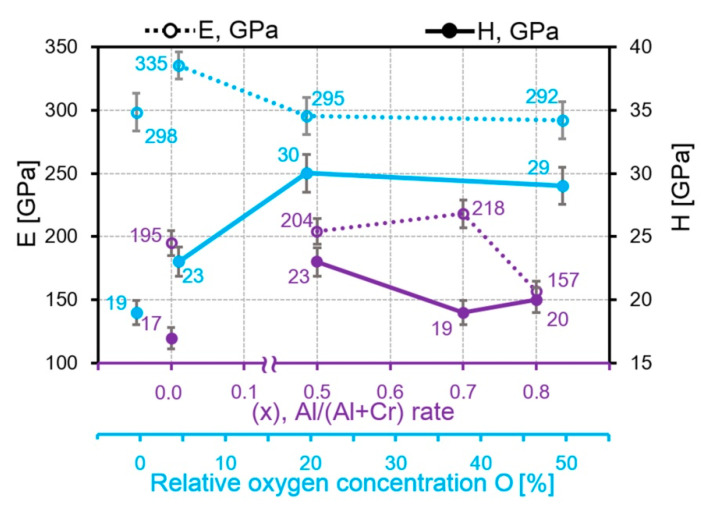
The dependences of *E* and *H* from the aluminum content in the AlCrN coatings and on the oxygen content in the CrON coatings.

In addition to roughness and microparticle amount (subsequently transforming into a modified material), one should take into account the mechanical properties of the “smooth” surface of the coatings when evaluating the tribological properties of coatings. The elastic modulus *E* at the depth of up to 100 nm for the CrON coatings is 292–335 GPa, which is higher compared to 157–204 GPa for AlCrN (Figure 14). For the AlCrN coatings, with the increase in the Al content till 80%, *E* decreases, which is well traced on the “load–depth” curves, which, with the Al content increase, gradually shifts to the right (Figure S3a). According to the curves, the CrON coatings are divided into two groups, CrN and CrO(50)N are referred to as coatings with “low *E*” and CrO(5)N and CrO(20)N as coatings with “high *E*”. According to the shape of the “load–depth” curves (the part of plastic deformation), we can conclude that the CrON coatings are more plastic than AlCrN (Figure S3b). These curves were obtained on a “smooth” surface at a penetration depth of up to 110 nm.

**Figure 15:**
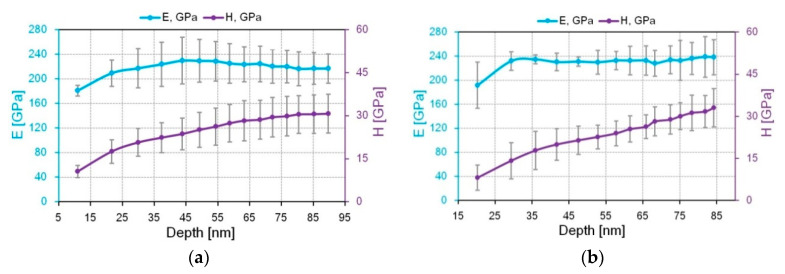
The dependences of *E* and *H* from the indentation depth in CrN (**a**) and Al_50_Cr_50_N coatings (**b**).

The dependences of *E* and *H* on the indentation depth in the CrN and Al_50_Cr_50_N coatings are shown in Figure 15. For the NI progressive partial load/unload curves with partial unloading of the CrN and Al_50_Cr_50_N coatings used for the calculation of *E* and *H*, these dependences are shown in Figure S4. The value of H in the upper layer of the coatings is significantly lower than at the depth of 50–100 nm, where the values are stable. For the Al_50_Cr_50_N coating, this difference is more than four times (Figure 15).

**Figure 16:**
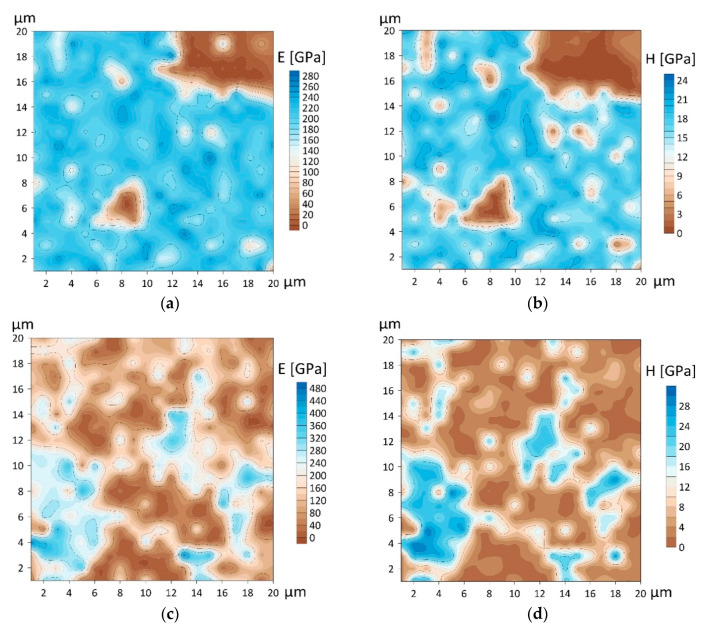
Elastic modulus (**a**,**c**) and microhardness (**b**,**d**) maps of CrN (**a**,**b**) and Al_50_Cr_50_N (**c**,**d**) coatings, area of 20 × 20 μm^2^.

To assess the distribution of mechanical properties over the surfaces of the coatings, maps of *E* and *H* were constructed, with an average indentation depth of around 20 nm (Figure 16). The maps fit well the morphology of the coatings (Figure 4a,b). The softer areas on the maps, marked in brown, refer to the microparticles. They clearly demonstrate an increase in the number of softer particles in the AlCrN coatings and their distribution over the surface (Figure 16). The average *E* values of the CrN coating obtained with TINA system were 180 ± 69 GPa and for *H* were 14.4 ± 6.6 GPa. The average values of *E* for the Al_50_Cr_50_N coating were 142 ± 109 GPa and for *H* were 8.2 ± 8.0 GPa.

The larger microparticle volumes on the AlCrN coating surface lead to the formation of a modified soft layer thicker which was than that of the CrON coatings [33] (Figure S3). The presence of such a layer can reduce friction [33]. However, it is impossible to draw a full analogy with lubrication without taking into account the properties of the hard underlying layers of the wear-resistant coating. For example, in the case of the elastohydrodynamic lubricant, a reduction in the friction coefficient with the increasing coating thickness was mentioned in some theoretical studies. Thus, according to [67], in the case of sliding contact, the increasing soft coating thickness leads to a reduction in both normal and tangential stresses; correspondingly, the friction force will decrease. In this case, a thinner modified layer of microparticles with a harder CrON coating showed lower values of the friction coefficient and the forces than in the case of a thicker layer of the AlCrN coating. The microstructure of the “smooth” coating surface with smaller cells in CrON than in AlCrN coatings also played a role, which helped to distribute evenly and retain the modified layer.

The influence degree of the microparticle geometry and h-AlN, c-CrN phase intensity on the friction coefficient was shown by the correlation coefficients (Figures S4 and S5, Tables S1–S4).

For all geometrical characteristics of particles (*d*, *h*, *S*, *V*, *Ra*), high correlations (*r* > 0.7) with *Cfr* were found. For *h* and *V*, the correlation coefficient of the CrON coatings was especially high—0.84 and −0.90. This result indicates a significant effect of *h* and *V* of the microparticles on *Cfr* and that, along with *E*, the geometric characteristics of the coating microparticles are significant features of the friction process and can be used in analytical models to solve the problem of *Cfr* determination. Most of the correlations for the AlCrN coatings are positive, while for the CrON coatings, they are negative. This means that in order to reduce *Cfr* for coatings in the AlCrN group, *h*, *S*, *V*, *Ra* and *E* should be reduced, while for coatings in the CrON group, *E* should be reduced but *d*, *h*, *S*, *V*, *Ra* should be increased (Figures S6 and S7).

The use of the peak intensity of c-CrN and h-AlN phases in the coatings as a criterion for the correlation analysis gave stronger correlations between the geometrical characteristics of the microparticles and the friction coefficient (*r* > 0.9). The highest is obtained for *h* (−1.0) and *V* (−0.99) (Tables S1–S4).

## 4. Conclusions

Two sets of CrN-based coatings, one with the addition of aluminum and the other with the addition of oxygen, were deposited by cathodic arc evaporation. The effect of metal and non-metal addition on the geometry of surface defects, i.e., diameter, height and volume of the microparticles, all above the coating surface, was assessed. The main findings are as follows:

(1) Strong correlations with a correlation coefficient of 0.82–1.00 between the friction coefficient obtained under conditions of sliding friction without a lubricant and the geometric characteristics of the microparticles on the coating surface (content, roughness, diameter, height, area and volume) for AlCrN coatings with 50%, 70%, and 80% aluminum and CrON coatings containing 5%, 20% and 50% oxygen have been established.
(2) It was found that the friction coefficient does not change significantly with the increase in aluminum content but significantly decreases with the increase in the oxygen content.
(3) The roughness parameters decrease with the increase of the Al concentration in AlCrN. For the CrON coatings, the opposite effect is observed. Similar relationships are observed for the size of the microparticles and their height for both types of coating.

This allows us to consider the geometric characteristics of the microparticles as significant features in the problem of friction coefficient determination.

## Figures and Tables

**Table 1:** The parameters of the multistep coating preparation.

Parameter\Coating	AlCrN	CrON
Deposition system	TINA 900M [10] (Vakuumtechnik Dresden GmbH, Dresden, Germany)	BULAT 3T [30] (Kharkov Institute of Physics and Technology, Kharkiv, Ukraine)
Cathode	Cr, AlCr (50:50), (70:30) and (80:20)	Cr
Cathode diameter [mm]	100	60
Base pressure [Pa]	1 × 10^−3^	2 × 10^−3^
Cathode-substrate distance [mm]	180	300
Rotation [rev/min]	2	30
Ion etching		
Bias [V]	−600	−1300
Argon pressure [Pa]	0.5	0.5
Cr arc current [A]	80	90
Etching time [min]	10	3
Adhesion layer		
Type of the layer	Cr	Cr
Cathode current [A]	80	90
Argon pressure [Pa]	0.5	0.5
Deposition temperature [°C]	350	400
Bias [V]	−100	−100
Thickness [µm]	0.1	0.1
Proper layer		
Cathode current [A]	80	90
Total pressure [Pa]	3	1.8
Nitrogen pressure [Pa]	3	-
Relative oxygen concentration O_2(x)_ = O_2_/(N_2_ + O_2_)	-	0, 5, 20, 50%
Deposition temperature [°C]	350	400
Bias [V]	−100	−150
Thickness [µm]	3	3
Investigated coatings		
Amount	4	4
Composition	CrN, Al_50_Cr_50_N, Al_70_Cr_30_N, Al_80_Cr_20_N,	CrN, CrO(5)N, CrO(20)N, CrO(50)N

## Supplementary Materials

The following are available online at https://www.mdpi.com/2079-4991/10/12/2361/s1, Figure S1: SEM images and spectra of microparticles and surface in Al_70_Cr_30_N and CrO(5)N coatings, Figure S2: AFM image with the “layered” microstructure of microparticle from Figure 4a, Figure S3: NI complete load/unload curves in AlCrN coatings (a) and in CrON coatings (b), Figure S4: NI progressive partial load/unload curves with partial unloading of CrN (a) and Al_50_Cr_50_N (b) coatings, Figure S5: Upper modified layer formed under tribological load on the CrN (a) and CrO(50)N (b) coatings surface, area of 20 × 20 μm^2^, Figure S6: Correlation coefficients between *Cfr* and *E* and particles characteristics for AlCrN coatings, Figure S7: Correlation coefficients between *Cfr* and *E* and particles characteristics for CrON coatings, Table S1: Correlation between intensity of h-AlN (100) (101), c-CrN (200) and particles characteristics (areas 40 × 40 μm^2^ and 20 × 20 μm^2^), *E*, *H*, *Cfr* for AlCrN coatings, Table S2: Correlation between intensity of h-AlN (100) (101), c-CrN (200) and particle content, *E*, *H*, *Cfr* for AlCrN coatings, Table S3: Correlation between intensity of c-CrN (111), Cr_2_O_3_ (104) and particles characteristics (areas 40 × 40 μm^2^ and 20 × 20 μm^2^), *E*, *H*, *Cfr* for CrON coatings, Table S4: Correlation between intensity of c- CrN (111), Cr_2_O_3_ (104) and particle content, *E*, *H*, *Cfr* for CrON coatings.
