# Supplementary material for: Effect of Metallic or Non-Metallic Element Addition on Surface Topography and Mechanical Properties of CrN Coatings
Source: Nanomaterials (Basel). 2020 Nov 27;10(12):2361. doi: 10.3390/nano10122361 (PMC7761171; doi:10.3390/nano10122361)
Supplement: Supplementary file 1 [file nanomaterials-10-02361-s001.pdf]

Supplementary Materials

Effect of Metallic or Non-Metallic Element Addition on Surface Topography and Mechanical Properties of CrN Coatings

Tatyana Kuznetsova [1], Vasilina Lapitskaya [1], Anastasiya Khabarava [1], Sergei Chizhik [1], Bogdan Warcholinski [2], Adam Gilewicz [2], Aleksander Kuprin [3], Sergei Aizikovich [4,*] and Boris Mitrin [4]

[1] Nanoprocesses and Technology Laboratory, A.V. Luikov Institute of Heat and Mass Transfer of National Academy of Science of Belarus, 15, P. Brovki str., 220072 Minsk, Belarus; kuzn06@mail.ru (T.K.); vasilinka.92@mail.ru (V.L.); av.khabarova@mail.ru (A.K.); chizhik_sa@tut.by (S.C.)

[2] Faculty of Mechanical Engineering, Koszalin University of Technology, 2, Śniadeckich, 75-453 Koszalin, Poland; bogdan.warcholinski@tu.koszalin.pl (B.W.); adam.gilewicz@tu.koszalin.pl (A.G.)

[3] National Science Center Kharkov Institute of Physics and Technology, 1, Academic str, 1., 61108 Kharkiv, Ukraine; kuprin@kipt.kharkov.ua

[4] Research and Education Center "Materials", Don State Technical University, 1, Gagarin sq., 344003 Rostov-on-Don, Russia; bmitrin@dstu.edu.ru

* Correspondence: s.aizikovich@sci.donstu.ru; Tel.: +7-863-238-15-58

Received: 20 November 2020; Accepted: 25 November 2020; Published: date

---

Figure S1 shows SEM images and spectra of microparticles and surface in $Al_{70}Cr_{30}N$ and CrO(5)N coatings.

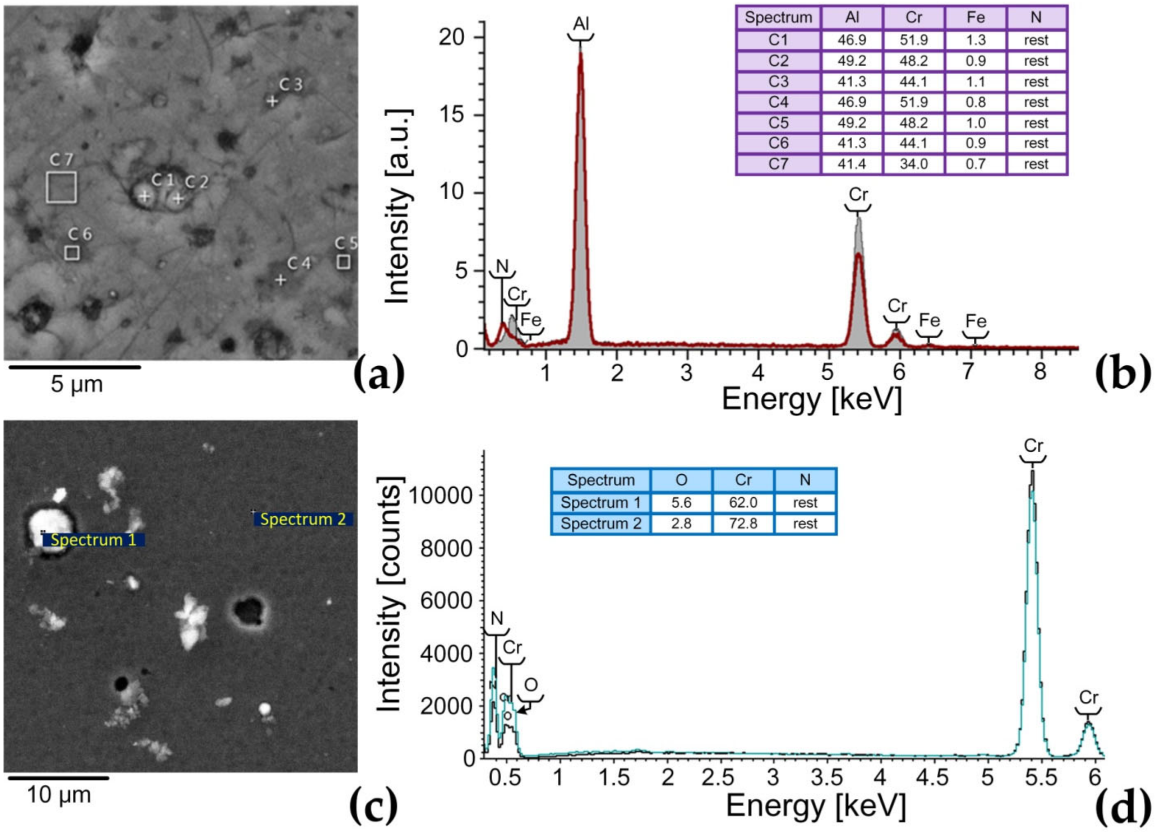

Figure S1. SEM images and spectra of microparticles and surface in $Al_{70}Cr_{30}N$ and CrO(5)N coatings: (**a**,**c**): image; (**b**,**d**): spectrum and table with the elements content; (**a**):$Al_{70}Cr_{30}N$, ×10000; (**d**): CrO(5)N, ×5000.

AFM image of microparticle with the «layered» microstructure is shown in Figure S2.

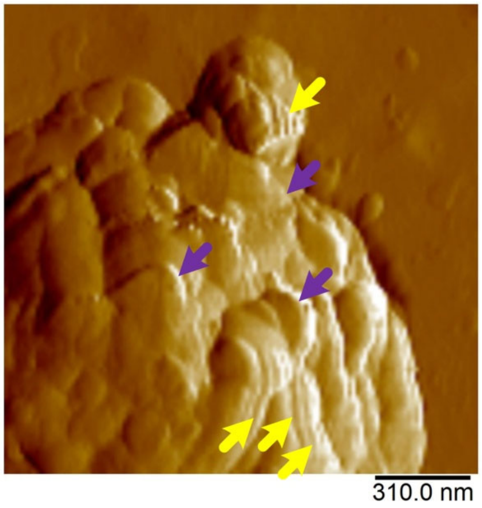

Figure S2. AFM image with the layered microstructure of microparticle from Figure 4a. Examples of layers are marked with violet; sublayers are marked with yellow.

The NI load/unload curves of CrN and $Al_{50}Cr_{50}N$ coatings are shown in Figures S3, S4.

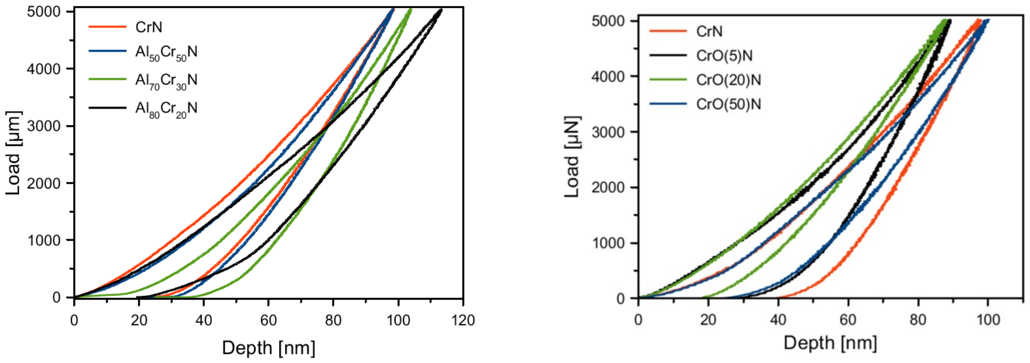

(**a**) (**b**)

Figure S3. The NI completely load/unload curves in AlCrN coatings (**a**) and in CrON coatings (**b**).

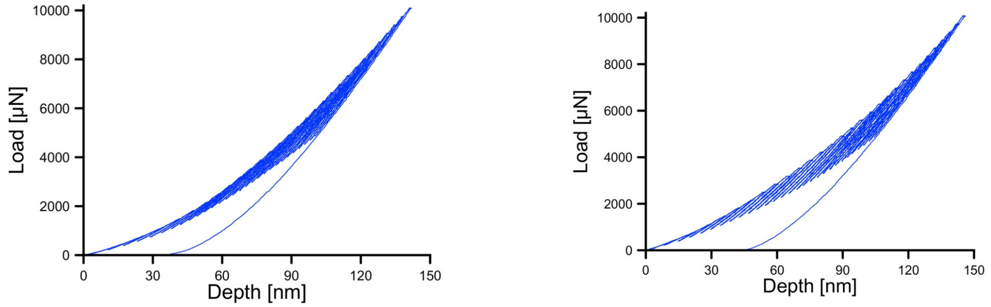

(**a**) (**b**)

Figure S4. The NI progressive partial load/unload curves with partial unloading of CrN (**a**) and $Al_{50}Cr_{50}N$ (**b**) coatings.

Figure S5. shows the upper modified layer formed under tribological load on the CrN and CrO(50)N coatings surface.

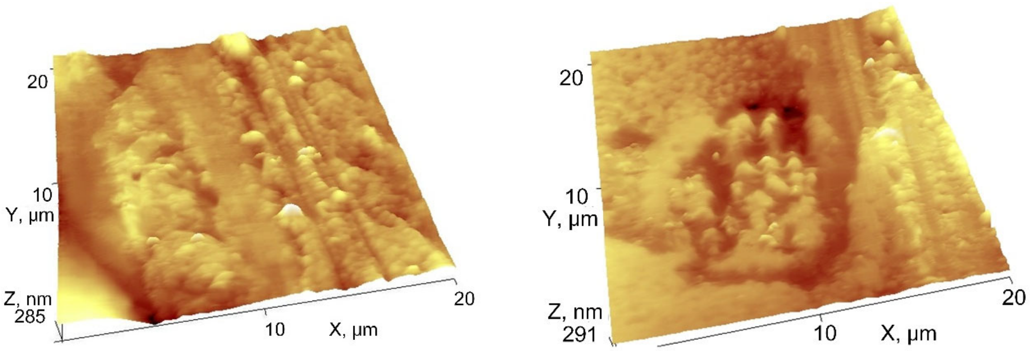

(**a**) (**b**)

Figure S5. The upper modified layer formed under tribological load on the CrN (a) and CrO(50)N (b) coatings surface, area of 20 × 20 μm^2.

The correlation coefficients between *Cfr* and *E* and particles characteristics for AlCrN coatings are shown in Figure S6.

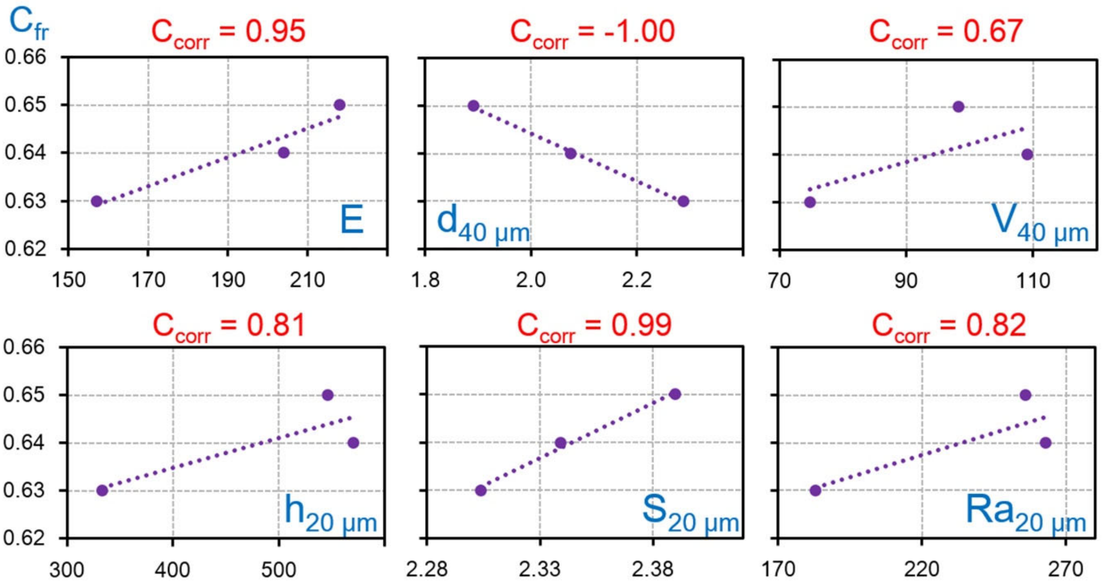

Figure S6. The correlation coefficients between *Cfr* and *E* and particles characteristics for AlCrN coatings.

The correlation coefficients between *Cfr* and *E* and particles characteristics for CrON coatings are shown in Figure S7.

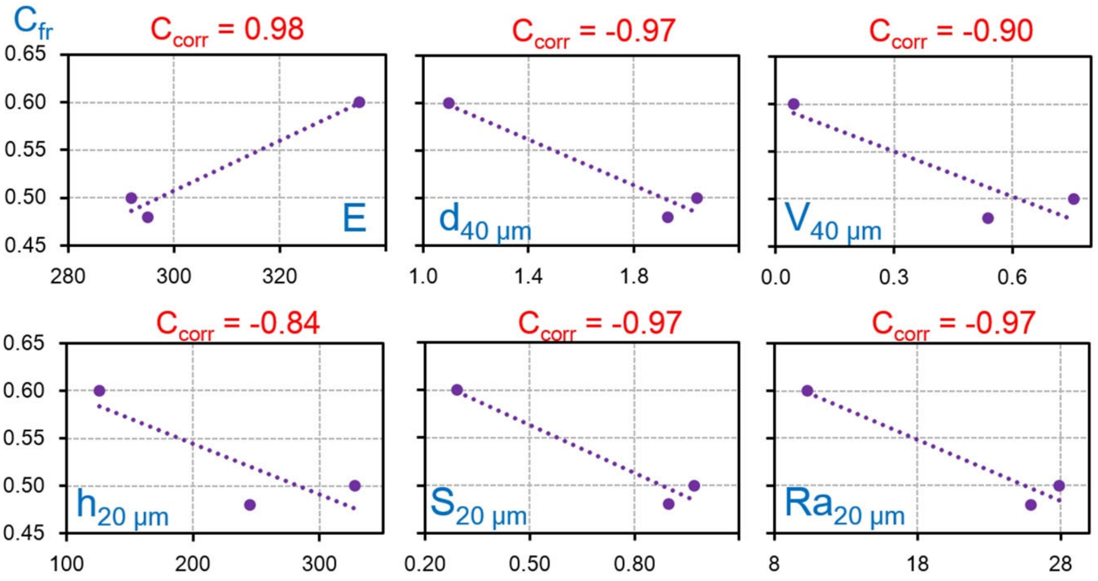

Figure S7. The correlation coefficients between *Cfr* and *E* and particles characteristics for CrON coatings.

Tables S1–S4 contain the initial dates for calculation and the correlation coefficients for $Al_{70}Cr_{30}N$ and CrO(5)N coatings.

Table S1. The correlation between intensity of h-AlN (100) (101), c-CrN (200) and particles characteristics (areas 40 × 40 μm^2 and 20 × 20 μm^2), E, H, Cfr for AlCrN coatings.

Characteristic	Coating			Correlation coefficient	
	$Al_{50}Cr_{50}N$	$Al_{70}Cr_{30}N$	$Al_{80}Cr_{20}N$	C_{corr}	C_{corr}

**Intensity of h-AlN (100) (101), [cps]**		123	1165	2372	(h-AlN)	(c-CrN)
Intensity of c-CrN (200), [cps]		546	850	1068		
d, [μm]	40 × 40 μm^2	2.1	1.9	2.3	0.57	0.46
h, [nm]		845	690	547	**−1.00**	**−1.00**
S, [μm^2]		4.86	5.01	5.95	**0.94**	**0.88**
V, [μm^3]		109.05	98.20	74.76	**−0.99**	**−0.95**
Ra, [nm]		272	265	241	**−0.97**	**−0.92**
Rq, [nm]		413	359	373	−0.68	**−0.78**
d, [μm]	20 × 20 μm^2	1.4	1.4	1.4	**0.98**	**0.95**
h, [nm]		571	546	333	**−0.93**	**−0.87**
S, [μm^2]		2.34	2.39	2.30	−0.44	−0.32
V, [μm^3]		17.7	14.3	10.0	**−1.00**	**−0.99**
Ra, [nm]		263	256	183	**−0.92**	**−0.86**
Rq, [nm]		376	341	241	**−0.97**	**−0.93**

Table S2. The correlation between intensity of h-AlN (100) (101), c-CrN (200) and particle content, E, H, C_{fr} for AlCrN coatings.

**Characteristic**	**Coating**			**Correlation coefficient**	
	**$Al_{50}Cr_{50}N$**	**$Al_{70}Cr_{30}N$**	**$Al_{80}Cr_{20}N$**	**C_{corr} (h-AlN)**	**C_{corr} (c-CrN)**
Intensity of h-AlN (100) (101), [cps]	123	1165	2372		
Intensity of c-CrN (200), [cps]	546	850	1068		
Particle content, % (40 × 40 μm^2)	11.5	31.6	26.8	**0.70**	**0.79**
Particle content, % (20 × 20 μm^2)	25.7	27.5	25.9	0.06	0.19
E, GPa	204	218	157	**−0.76**	−0.67
H, GPa	23	19	20	−0.69	**−0.78**
C_{fr}	0.64	0.65	0.63	−0.54	−0.42

Table S3. The correlation between intensity of c-CrN (111), Cr_2O_3 (104) and particles characteristics (areas 40×40 μm^2 and 20×20 μm^2), E, H, C_{fr} for CrON coatings.

**Characteristic**		**Coating**			**Correlation coefficient**	
		CrO(5)N	CrO(20)N	CrO(50)N	C_{corr} (CrN)	C_{corr} (Cr_2O_3)
**Intensity of c-CrN (111), [cps]**		5386	386	434		
Intensity of Cr_2O_3 (104), [cps]		401	399	568		
d, [μm]	40 × 40 μm^2	1.1	1.9	2.0	**−0.99**	0.58
h, [nm]		192	418	504	**−0.96**	0.71
S, [μm^2]		1.09	3.68	4.51	**−0.97**	0.68
V, [μm^3]		0.04	0.54	0.76	**−0.95**	0.73
Ra, [nm]		4	40	48	**−0.98**	0.63
Rq, [nm]		17	85	98	**−0.99**	0.62
d, [μm]	20 × 20 μm^2	0.8	1.4	1.5	**−0.97**	0.68
h, [nm]		126	245	328	**−0.91**	**0.80**
S, [μm^2]		0.29	0.90	0.97	**−0.99**	0.58
V, [μm^3]		0.01	0.04	0.14	−0.69	**0.97**
Ra, [nm]		10	26	28	**−0.99**	0.58
Rq, [nm]		16	49	53	**−1.00**	0.56

Table S4. The correlation between intensity of c- CrN (111), Cr_2O_3 (104) and particle content, E, H, C_{fr} for CrON coatings.

Characteristic	Coating			Correlation coefficient	
	CrO(5)N	CrO(20)N	CrO(50)N	C_{corr} (CrN)	C_{corr} (Cr_2O_3)
Intensity of c-CrN (111), [cps]	5386	386	434		
Intensityof c- Cr_2O_3 (104), [cps]	401	399	568		
Particle content, % (40 × 40 μm²)	0.9	3.0	5.1	**−0.86**	**0.86**
Particle content, % (20 × 20 μm²)	1.8	2.0	3.2	−0.64	**0.98**
E, GPa	335	295	292	**1.00**	−0.54
H, GPa	23	30	29	**−0.99**	0.37
C_{fr}	0.60	0.48	0.50	**0.99**	−0.35
